# 晚期非小细胞肺癌维持治疗的现状与展望

**DOI:** 10.3779/j.issn.1009-3419.2010.06.013

**Published:** 2010-06-20

**Authors:** 琳 权

**Affiliations:** 1 210029 南京，南京医科大学第一附属医院肿瘤科 Department of Oncology, the First Affiliated Hospital of Nanjing Medical University, Jiangsu Province Hospital, Nanjing 210029, China; 2 210029 南京，南京市胸科医院呼吸科 Department of Respiratiory Medicine, Nanjing Chest Hospital, Nanjing 210029, China

目前，非小细胞肺癌（non-small cell lung cancer, NSCLC）仍然是人类死亡率最高的恶性肿瘤之一，NSCLC按照发病率依次为腺癌、鳞癌、大细胞癌和非特指类型。对于大多数NSCLC患者，目前一线治疗方案是以铂类为基础的两药联合化疗或者针对特定人群使用分子靶向性药物，虽然大多数患者经过前期诱导治疗可以较为明显的改善临床症状和生活质量，但是大多数患者会在随后的1年内复发，1年生存率只有30%-50%^[1]^。维持治疗是指在诱导化疗结束后，患者对于诱导治疗有效，并且疾病处于相对稳定的情况下，继续使用没有交叉耐药并且副作用相对较小的药物进行治疗。目前对于NSCLC维持治疗的临床价值和治疗方案没有统一的认识，但是由于其有可能延缓NSCLC的复发，延长患者生存期，因此逐渐得到重视，尤其是近年来分子靶向性药物逐步应用于临床，使得NSCLC的维持治疗进一步拓展。

## 1 NSCLC维持治疗方案选择的原则

用于NSCLC维持治疗的药物较多，方案也互不相同，但是大多数学者认为选择和诱导治疗不同的药物或治疗方案可能具有更多的优势。恶性肿瘤患者体内对于化疗耐药的细胞数量会随着疾病发展不断增加和累积，因此早期使用没有交叉耐药的药物（治疗方案）可以在肿瘤细胞发生耐药前将其杀灭^[2, 3]^，从而阻止或者延缓临床耐药的发生。而且，近年来第三代细胞毒性药物、分子靶向性药物和免疫治疗逐步应用于临床，由于该类治疗方案较传统化疗药物副作用明显减低，因此可以较为长期的使用，提示其可能在未来的NSCLC维持治疗中扮演重要角色；同时维持治疗药物理论上可以延续和提高诱导治疗对于肿瘤细胞生长和增殖的抑制作用，进而提高无疾病进展生存期（progression-free survival, PFS）和总生存期（overall survival, OS）。因此使用没有交叉耐药并且副作用较小的单药或者联合治疗方案进行维持治疗可使NSCLC患者进一步获益。

## 2 基于化疗药物的维持治疗

### 2.1 培美曲塞

培美曲塞是一种多靶点的抗叶酸代谢复合物，目前同时被美国和欧盟批准用于晚期NSCLC的二线治疗以及联合顺铂用于非鳞癌的一线治疗^[4]^。由于培美曲塞本身的毒副作用较轻，患者耐受性较好，使其可能成为NSCLC维持治疗药物之一。2009年Belani^[5]^在ASCO年会上报告了一项Ⅲ期、随机双盲的临床试验，旨在验证培美曲塞作为维持治疗NSCLC的临床疗效，结果显示利用培美曲塞作为NSCLC诱导化疗后的维持治疗较安慰剂组可以明显改善NSCLC患者的PFS和OS，这项临床研究设计为：诱导治疗采用不含培美曲塞的铂类两药联合方案治疗4个疗程，然后将对于诱导治疗有效的患者随机分为培美曲塞维持治疗组和安慰剂组。分析结果发现使用培美曲塞进行维持治疗的患者PFS显著延长（4.04个月*vs* 1.97个月，*P* < 0.001）。其中非鳞癌的NSCLC患者可以从培美曲塞维持治疗中获益，生存期显著延长（15.5个月*vs* 10.3个月，*P*=0.002）。2009年Ciuleanu等^[6]^开展的一项多中心随机双盲的临床Ⅲ期试验，共纳入663例Ⅲb或者Ⅳ期的NSCLC患者，使用4个疗程以铂类为主的诱导方案后，对治疗有效者随机分为培美曲塞维持治疗组和安慰剂对照组，分析结果显示使用培美曲塞进行维持治疗的患者PFS和OS均显著延长（4.3个月*vs* 2.6个月；13.4个月*vs* 10.6个月），虽然培美曲塞组的药物相关副作用较多，但是经过对症治疗后均可以得到及时控制，未出现治疗相关死亡。通过以上两项较大规模的Ⅲ期临床试验目前可以得到以下结果：①单药培美曲塞作为维持治疗可以显著延长NSCLC患者，尤其是非鳞癌患者的PFS和OS；②对于鳞癌患者的效果不甚显著；③培美曲塞副作用较小，并且大多数为轻度可控。基于此临床试验，美国FDA于2009年7月批准培美曲塞作为维持治疗药物应用于NSCLC，尤其是非鳞癌的患者。

### 2.2 吉西他滨

2006年Brodowicz等^[7]^开展了一项Ⅲ期临床试验探索吉西他滨在NSCLC维持治疗中的价值，纳入该临床研究的患者首先使用顺铂+吉西他滨的联合化疗方案4个疗程，然后随机分为利用吉西他滨作为维持治疗组和对照组。虽然维持治疗组和对照组在治疗总反应率和反应时间上没有明显差异，但是吉西他滨维持治疗组的中位肿瘤进展时间（time to progression, TTP）显著延长（6.6个月*vs* 5个月；*P* < 0.001），维持治疗组的总生存有提高的趋势，但是没有达到统计学差异（13个月*vs* 11个月；*P*=0.19）。虽然吉西他滨组的副作用并不严重，但是维持治疗组仍有较多患者需要接受血制品输注（20% *vs* 6.3%; *P*=0.018），此外还需要注意的是这项临床试验中有16%的患者由于各种原因退出了该研究，同时吉西他滨维持治疗组有22%的患者由于各种原因延迟了治疗，虽然存在诸多不足，但是通过该临床研究依然可见吉西他滨作为NSCLC单药维持治疗的可行性和相对临床安全性。

### 2.3 紫杉醇和多西紫杉醇

两项临床研究^[8, 9]^涉及了紫杉醇作为NSCLC维持治疗的价值，结果在使用顺铂+紫杉醇4个疗程后利用紫杉醇维持治疗可以提高TTP和中位OS，并且耐受性较好，但是需要注意的是这两个临床试验的主要目的都不是评估紫杉醇单药在NSCLC维持治疗中的作用，因此在试验设计上可能并不十分完善，尚需进一步的Ⅲ期临床试验验证紫杉醇在维持治疗中的作用。多西紫杉醇（多西他赛）目前被批准应用于晚期NSCLC的一线和二线治疗，2008年Pino等^[10]^通过研究发现老年NSCLC患者（年龄>65岁）诱导治疗后使用紫杉醇进行维持治疗具有一定的临床疗效并且药物相关副作用较小，大多数患者可以较好的耐受维持治疗。2009年Fidias等^[11]^进行了一项旨在评价多西紫杉醇在NSCLC常规化疗后单药维持治疗的价值和安全性的临床试验，患者接受常规顺铂+吉西他滨联合化疗4个疗程后随机分为多西他赛维持治疗组和多西他赛挽救治疗组（待疾病进展后进行治疗），结果提示使用多西紫杉醇作为维持治疗的患者PFS和治疗总反应率显著提高，总体生存较对照组虽有提高，但没有统计学差异（11.7个月*vs* 9.1个月），毒副作用在两组间没有差异。以上临床试验表明紫杉醇作为维持治疗药物治疗NSCLC患者较为安全，尤其对于老年NSCLC患者，耐受性较好，可以明显提高治疗反应和PFS，但是对于OS的影响尚不明确。

### 2.4 长春瑞滨

长春瑞滨是一种通过抑制细胞有丝分裂来抑制和杀灭肿瘤细胞的化疗药物，应用于包括NSCLC在内的多种实体瘤和血液系统肿瘤。2005年Westeel等^[12]^利用丝裂霉素+异环磷酰胺+顺铂诱导治疗NSCLC后随机分为长春瑞滨维持治疗组和对照组以评估长春瑞滨单药维持治疗的安全性和有效性，试验结果显示无论在PFS还是OS，利用长春瑞滨维持治疗都没有显示其优越性，并且长春瑞滨治疗组治疗相关的毒副作用较大。

### 2.5 一线诱导化疗方案

对于诱导方案有效的患者，继续进行诱导方案治疗似乎是一个合理的维持治疗策略，但是多项临床研究均表明增加诱导治疗的疗程数不能使NSCLC患者进一步受益（生活质量、PFS和OS），同时随着化疗数的增加，化疗相关的副作用不断累积。2004年ASCO发布的NSCLC临床诊治指南^[13]^中推荐NSCLC患者对于诱导治疗有效患者不超过6个疗程，而无效患者不超过4个疗程。2010年NCCN指南^[14]^推荐晚期NSCLC进行4个-6个疗程的一线诱导治疗。基于ASCO和NCCN的指南推荐可以看出不断增加诱导化疗的疗程数（延续诱导治疗作为维持治疗）并不能使患者获得更多的临床受益反而增加毒性，同时随着一些新型治疗药物，尤其是分子靶向性药物的出现（吉非替尼、厄洛替尼和贝伐单抗等）很可能使推荐诱导治疗数进一步从6个疗程缩短为4个疗程，使得部分患者尽早过度到维持治疗阶段，在保证疾病疗效的同时，减少治疗相关的副作用。

## 3 基于分子靶向性药物的维持治疗

### 3.1 EGFR抑制剂

表皮生长因子受体（epidermal growth factor receptor，EGFR，亦称HER1或Erb-B1）是跨膜糖蛋白受体Erb-B家族成员之一，表达于80%的NSCLC表面，研究^[15]^发现表达此蛋白的患者预后较差。EGFR和配体EGF（或TGF-α）结合后可以触发下游的分子信号转导途径STAT、Ras-MAPK和PI3K/Akt等，从而促进肿瘤的生长、增殖、抗凋亡、转移和血管新生的能力。目前临床使用的EGFR抑制剂主要有吉非替尼、厄洛替尼和西妥昔单抗。2005年TRIBUTE是一个Ⅲ期临床试验，旨在验证常规化疗后使用厄洛替尼单药维持治疗的有效性。在TRIBUTE试验^[16]^中，预期生存超过4个月的861例NSCLC患者在接受常规铂类为主的化疗方案后随机分组（其中厄洛替尼维持治疗组408例，安慰剂对照组453例）。中位OS在两组患者中分别为13.6个月*vs* 12.2个月（*P*=0.04）；在740例存活超过6个月的患者中（厄洛替尼维持治疗组348例，安慰剂组392例），中位生存期为15.4个月*vs* 13.8个月（*P*=0.007）；2009年Cappuzzo等^[17]^在ASCO年会上报告了SATURN临床试验的最新结果，该试验为验证厄洛替尼作为单药维持治疗NSCLC的Ⅲ期临床试验，初步结果显示使用厄洛替尼进行维持治疗较安慰剂组可以显著提高患者的PFS（HR=0.71, 95%CI: 0.62-0.82, *P* < 0.000 1），并且厄洛替尼维持治疗组的12周疾病控制率也显著较高，由于该临床试验尚未结束，因此OS的结果尚未得出。2008年来自于日本West Japan Thoracic Oncology Group的Ⅲ期临床试验^[18]^，利用吉非替尼治疗经过常规化疗的晚期NSCLC患者，使用吉非替尼进行维持治疗的患者PFS显著延长，但是OS没有达到统计学差异。而进一步的亚组分析中发现对于病理类型为腺癌的患者，吉非替尼维持治疗可以显著延长PFS和OS，并且有统计学差异。本科室的小规模临床研究也发现中国成人肺腺癌患者常规化疗后，利用吉非替尼单药作为维持治疗，和对照组（随访观察）相比，使用吉非替尼作为维持治疗的肺腺癌患者的PFS和OS均明显延长，并且患者耐受性良好，副作用可控（数据未发表），为吉非替尼应用于选定NSCLC人群（东亚人种、肺腺癌、女性和非吸烟患者）提供了依据。2008年Pirker等^[19]^利用西妥昔单抗联合化疗作为NSCLC诱导和维持治疗，这些患者使用顺铂+长春瑞滨+西妥昔单抗进行诱导治疗后，单药西妥昔单抗进行维持治疗直到疾病进展，相对于化疗利用西妥昔单抗进行维持治疗的患者在OS有一定的提高，但是由于该研究维持治疗只使用了4周西妥昔单抗治疗，因此结果尚需进一步验证。以上多项临床研究结果显示EGFR酪氨酸激酶抑制剂作为NSCLC维持治疗中的肯定疗效，并且基础研究表明EGFR突变阳性患者对于EGFR TKI的治疗反应明显优于EGFR阴性患者，而EGFR的突变率在东亚人种、肺腺癌、女性和非吸烟患者中明显较高。因此提示我们对于选择性人群（东亚人种、肺腺癌、女性和非吸烟患者），使用EGFR抑制剂可能会取得更好的疗效，而这需要进一步的针对选择性人群的Ⅲ期前瞻性临床试验来验证EGFR抑制剂在维持治疗中的价值。

### 3.2 VEGF抑制剂

血管新生是肿瘤生长和转移重要的病理生理过程。肿瘤细胞分泌的各种促血管生成因子可以通过刺激促进肿瘤自身新生血管的生长，提供肿瘤局部需要的营养和氧气并及时清除局部的代谢产物，从而达到促进肿瘤生长的目的。血管新生过程中血管内皮细胞生长因子（vascular endothelial growth factor, VEGF）是最主要的促血管新生因子，并且基础研究已在包括NSCLC的多种肿瘤中证实了其重要性^[20]^。目前美国FDA已经通过利用VEGF拮抗剂治疗肺癌、结肠癌和肾癌，并且一些临床试验表明VEGF和传统化疗存在一定协同作用：可能通过改变肿瘤微环境局部的异常血管结构，增加局部化疗药物的浓度来增加化疗药物的敏感性。贝伐单抗是和VEGF具有高度亲和力的单克隆抗体。前期的临床试验表明联合贝伐单抗和传统化疗治疗晚期肺癌较单纯化疗组在反应率（response rate, RR）和PFS等方面具有一定的优势，但是对于OS改善不显著，同时发现在使用贝伐单抗治疗的鳞癌患者中发生咯血的危险性较高。基于以上试验的前期结果，ECOG进行了Ⅲ期临床试验（E4599）比较了卡铂+紫杉醇±贝伐单抗治疗878位晚期非鳞癌的NSCLC患者，加用贝伐单抗组继续使用贝伐单抗进行维持治疗直到疾病进展，结果显示联合治疗组在RR（*P* < 0.001）、PFS（HR=0.66, *P* < 0.001）和OS（HR=0.79, *P*=0.003)等指标上具有明显的优势。其后2009年的AVAiL Ⅲ期临床试验^[21]^比较了顺铂+吉西他滨±贝伐单抗（7.5 mg/kg或15 mg/kg）治疗晚期NSCLC，加用贝伐单抗组随后使用贝伐单抗进行维持治疗，AVAiL结果提示联合贝伐单抗作为NSCLC诱导和维持治疗在PFS和RR具有明显的优势，但是OS没有显著改善，并且贝伐单抗的两个剂量组疗效类似。E4599和AVAiLi两组临床试验在OS上的差异，可能的原因为紫杉醇可以诱导和动员循环内皮祖细胞（circulating endothelial precursors, CEPs）而此时加用VEGF抑制剂可能发挥协同作用，而吉西他滨却没有类似的作用。最新的2009年ASCO年会上Miller等^[22]^报告了ATLAS Ⅲ期临床试验的结果，其目的在于验证联合贝伐单抗+厄洛替尼和单药贝伐单抗在NSCLC诱导化疗后维持治疗中的作用，该试验共纳入1 160例患者，诱导化疗后随机分组，结果显示联合贝伐单抗+厄洛替尼组的PFS优于单药贝伐单抗组（4.8个月*vs* 3.7个月，*P*=0.001 2）；同时2009年美国西南癌症协作组（Southwest Oncology Group, SWOG）开展的Ⅲ期临床试验^[23]^，使用贝伐单抗+西妥昔单抗+卡铂+紫杉醇进行诱导治疗后，继续使用贝伐单抗+西妥昔单抗作为维持治疗，试验共纳入110例NSCLC患者，中位随访15个月后结果显示疾病控制率为77%，中位随访时间为15个月，PFS为7个月，总生存为14个月，1年生存率为57%，并且毒副作用未有增加，以上临床试验进一步验证了一线诱导方案和维持方案中引入贝伐单抗+西妥昔单抗联合靶向性治疗药物的安全性和临床疗效。

## 4 基于免疫治疗的维持治疗

理论上给NSCLC患者免疫针对肿瘤特异性抗原的治疗性疫苗可以使此类患者获得针对肿瘤细胞较为特异性的免疫力，从而达到靶向性杀伤肿瘤细胞的目的。但是早期对于肺癌患者的疫苗等免疫相关研究没有取得明显进展。近年来得益于肿瘤免疫学的快速发展，关于针对NSCLC特异性抗原的疫苗研究领域取得了长足的进展，试验的结果显示此类新型疫苗对于NSCLC患者具有一定的临床疗效，并且几乎没有治疗相关的毒副作用^[24]^。在这其中，针对肿瘤细胞表面粘蛋白分子1（mucin 1, MUC1）的脂质体疫苗L-BLP25已经进入人体临床试验，MUC1分子是表达在上皮细胞表面的跨膜蛋白，虽然其确切作用尚不十分明确，但是基础研究表明MUC1的异常表达是实体肿瘤患者预后较差的指标之一，在这项随机的Ⅱb期临床试验中，将经过诱导治疗的Ⅲ/Ⅳ期NSCLC患者随机分为L-BLP25疫苗治疗组和支持治疗组，结果显示疫苗治疗几乎所有病人（96.6%）都可以顺利完成所有接种过程，中位生存期分别为17.4个月和13个月，目前更大规模的使用L-BLP25进行维持治疗的Ⅲ期多中心临床试验（START试验）正在进行；Belagenpumatucel-L是一种经过基因改造、可以持续分泌针对TGF-β2反义寡核苷酸的疫苗，而TGF-β2是一种免疫抑制因子，是NSCLC预后不良的指标。一项随机Ⅱ期临床试验共纳入75例Ⅱ-Ⅳ期的NSCLC患者，常规诱导治疗后进行3个剂量组的维持治疗，结果表明接受高剂量组维持治疗的患者平均生存为581天，而最低剂量组只有252天^[25]^，目前基于疫苗的临床Ⅲ期（STOP试验）试验已经展开，预计结果将会在2011年公布。

## 5 小结和展望

目前多数NSCLC患者的治疗策略仍然为以铂类为主的一线方案治疗4个-6个疗程后进行随访观察直至疾病进展，但是由于缺乏有效的维持和巩固治疗，因此大多数患者在治疗结束后4个月-6个月疾病复发。近年来对于NSCLC维持治疗的不断探索取得了令人鼓舞的结果，包括第三代化疗药物、分子靶向药物和疫苗等免疫治疗等多种治疗手段不断引入临床，在提高NSCLC患者PFS和/或OS的同时，较为明显的降低了治疗相关的副作用。目前已有多项临床Ⅱ/Ⅲ期试验表明维持治疗在NSCLC中的具有较为重要的地位，虽然大多数临床试验结果表明维持治疗可以明显改善RR和PFS，但是对于OS的改善尚不明确和统一，在这其中关于培美曲塞、吉非替尼和厄洛替尼的多项临床试验结果表明这几种药物作为单药维持治疗不但可以改善患者PFS，而且可以延长OS。与此同时通过技术手段检测不同指标预测疗效也成为NSCLC维持治疗的热点。比如Ciuleanu等开展的关于培美曲塞维持治疗的临床试验显示非选择性整体NSCLC患者的平均生存获益只有2.8个月，但是进一步亚组分析发现组织起源为非鳞癌的NSCLC患者平均生存达到5.2个月，几乎提高近一倍；再如对于利用吉非替尼进行治疗的患者，如果分析整体非选择性NSCLC患者人群较难发现吉非替尼相对于化疗药物的优越性，但是通过检测发现具有EGFR突变的患者，其疗效明显优于不具有EGFR突变的患者。因此通过利用免疫组化、FISH或DNA测序等手段检测可以预测治疗疗效的相关治疗可以使目前的维持治疗更加准确，进一步使得特定人群获益，而减少非敏感群体的治疗相关毒副作用和治疗花费。因此未来NSCLC维持治疗的方向，应该立足于肿瘤免疫学、分子生物学和临床医学的迅猛发展，同时结合基础科研和临床试验的成果，基于精确预测NSCLC维持治疗疗效指标的基础上针对不同受益群体不断优化维持治疗的治疗方案和疗程，不断完善个体化治疗。
